# A review of the trunk surface metrics used as Scoliosis and other deformities evaluation indices

**DOI:** 10.1186/1748-7161-5-12

**Published:** 2010-06-29

**Authors:** Petros Patias, Theodoros B Grivas, Angelos Kaspiris, Costas Aggouris, Evangelos Drakoutos

**Affiliations:** 1 School of Surveying Engineering, Aristotle University of Thessaloniki, GR-541 24 Thessaloniki, Greece; 2 Department of Trauma and Orthopaedics, "Tzanio" General Hospital of Piraeus - NHS, Tzani & Afendouli str, 18536, Piraeus, Greece; 3 Orthopaedic Surgeon, Department of Trauma and Orthopaedics, "Thriasio" General Hospital - NHS, G. Gennimata Av. 19600, Magoula, Attica, Greece

## Abstract

**Background:**

Although scoliosis is characterized by lateral deviation of the spine, a 3D deformation actually is responsible for geometric and morphologic changes in the trunk and rib cage. In a vast related medical literature, one can find quite a few scoliosis evaluation indices, which are based on back surface data and are generally measured along three planes. Regardless the large number of such indices, the literature is lacking a coherent presentation of the underlying metrics, the involved anatomic surface landmarks, the definition of planes and the definition of the related body axes. In addition, the long list of proposed scoliotic indices is rarely presented in cross-reference to each other. This creates a possibility of misunderstandings and sometimes irrational or even wrong use of these indices by the medical society.

**Materials and methods:**

It is hoped that the current work contributes in clearing up the issue and gives rise to innovative ideas on how to assess the surface metrics in scoliosis. In particular, this paper presents a thorough study on the scoliosis evaluation indices, proposed by the medical society.

**Results:**

More specifically, the referred indices are classified, according to the type of asymmetry they measure, according to the plane they refer to, according to the importance, and relevance or the level of scientific consensus they enjoy.

**Conclusions:**

Surface metrics have very little correlation to Cobb angle measurements. Indices measured on different planes do not correlate to each other. Different indices exhibit quite diverging characteristics in terms of observer-induced errors, accuracy, sensitivity and specificity. Complicated positioning of the patient and ambiguous anatomical landmarks are the major error sources, which cause observer variations. Principles that should be followed when an index is proposed are presented.

## Introduction

Our interest in the study of the trunk surface (TS) deformity is recently increased due to a variety of reasons.

The cosmetic improvement of the trunk after any treatment is of paramount importance to the child under treatment and his family. The TS symmetry is what it is seen and praised by them and not the radiograph itself which is traditionally used by the physician. TS symmetry is also one of the elements intergrading and improving the quality of life of patients, an issue vital for any human being [1]. This was actually the motivation behind both the development of a variety of devices for documentation and evaluation of TS shape and the creation of a variety of indices that are currently used to access the state of such deformities.

The concept is how to collect data related to TS on physiology, to document the pathology, to assess the effect on the TS deformity of any surgical or conservative treatment comparing the pro- to post-treatment state. The characterization of the threshold of normality to pathology is a complex issue that also needs investigation. Although not yet sensitive enough to detect small changes for monitoring of curve natural progression, TS analysis can help to document the external asymmetry associated with different types of spinal curves in scoliosis as well as the cosmetic improvement obtained after surgical interventions [2].

The review and the evaluation of the TS metrics used as Scoliosis or any deformity evaluation indices would be very useful and would offer some objective assessing tools for the interested physicians.

## Scoliosis screening practice

Scoliosis is a deformity of the spine in which there are one or more lateral curvatures deviating from the midline in the coronal plane. Although scoliosis is characterized by lateral deviation of the spine, a 3D deformation actually is responsible for geometric and morphologic changes in the trunk and rib cage [3].

The goal of scoliosis screening is to detect scoliosis at an early stage, when the deformity is likely to go unnoticed and there is an opportunity for a less invasive method of treatment, or less surgery, than would otherwise be the case. What in reality scoliosis school screening program does, using the scoliometer or any other surface measuring device, is reveal children with surface, mainly thoracic, deformity. It does not reveal the scoliosis per se. It is now definitely accepted that the surface deformity does not accurately predict the magnitude of scoliosis, especially in younger children. As Bunnell characteristically states [4] "it has become apparent from many reports that, although there is a significant correlation between clinical deformity and radiographic measurement, the standard deviation is so high that it is not possible to reliably predict the degree of curvature from surface topography in any given patient by any technique".

Traditionally, scoliosis screening is done either by Adam test or using other optical techniques, while the radiographic measurement of Cobb angle is considered the golden standard.

### The Adam test

The first step in the scoliosis examination is simple inspection. This includes inspection of a standing patient from behind and optical evaluation of asymmetries in shoulders, scapulae, waistline and the distance of the arms from the trunk, as well as the "balance" of the head.

**Figure 1:**
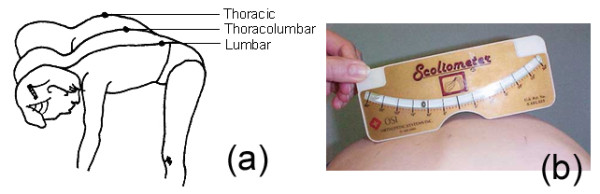
**(a) The Adams forward-bending test and (b) the "scoliometer"**.

The principal screening test for scoliosis is the physical examination of the back, which includes the Adams [5] forward-bending test (Fig. 1a), while the "scoliometer" (Fig. 1b) quantifies the trunk deformation. The bending test (Adams test) is performed in both standing and sitting forward bending positions. In the standing forward bending position, the examined person is asked to bend forward looking down, keeping the feet approximately 15 cm apart, knees braced back, shoulders loose and hands positioned in front of knees or shins with elbows straight and palms opposed. Any leg length inequality is not usually corrected. The scoliometer is used at three areas of interest: at upper thoracic (T3-T4), main thoracic (T5-T12) and at the thoraco-lumbar area (T12-L1 or L2-L3). In the sitting forward bending position, the examined person is seated on a chair (40 cm high) and is asked to bend forwards and place the head between the knees with the shoulders loose, elbows straight and hands positioned between knees. The scoliometer measurements are obtained successively at the same three areas of interest as in the standing forward bending position. Scoliometer measurement equal to 0° is defined as symmetry at the particular level of the trunk. Any other scoliometer value is defined as asymmetry [6].

It is reported that Adams test actually demonstrates the rotational component of scoliosis, since the rib prominence is the result of the ribcage rotating along with the spine [7]. The Adams test is considered a very sensitive clinical examination as compared to Cobb angle [8]. However, the sensitivity and specificity^1 ^varies depending upon the skills of the examiner, the location of the curve, and the magnitude of the curve [9]. The range of sensitivity and specificity of the forward bend test varying degrees of scoliosis have been reported as follows [10,9]:

▪ Thoracic scoliosis with Cobb angle ≥10° - sensitivity 74% - 84%, specificity 78%-93%

▪ Thoracic scoliosis with Cobb angle ≥20° - sensitivity: 92% - 100%, specificity 60% - 91%

▪ Lumbar scoliosis with Cobb angle ≥20° - sensitivity 73%, specificity: 68%

▪ Scoliosis with Cobb angle ≥40° - sensitivity 83%, specificity 99%

Sensitivity: the ability of a test to correctly identify patients with scoliosis. It is defined as follows:

High Sensitivity means low rate of false negatives, ie. the number of scoliotic patients classified as normal is small.

Specificity: the ability of a test to correctly identify patients without scoliosis. It is defined as follows:

High Specificity means low rate of false positives, ie. the number of normal patients classified as scoliotic is small.

It is very important to note that in younger children the concordance of the surface and spinal deformity is weak and it becomes stronger as the children are growing up. Therefore, in younger children with surface trunk asymmetry, the prediction of the spinal deformity alone from the surface topography is inaccurate, simply because surface topography reveals the thoracic cage and the spinal deformity together.

It has also been reported that, in typical screening settings where the prevalence and positive predictive value are relatively low, for every curve >10° detected, there are 1-5 false-positives; similarly, for every curve > 20° detected, there are 3-24 false-positives [11].

**Figure 2:**
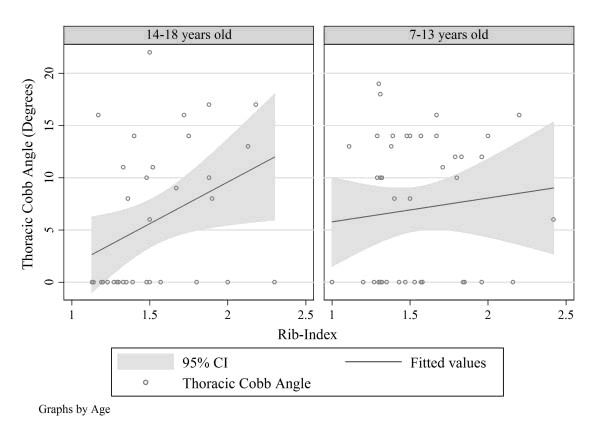
**Univariate linear regression models by age group**. Thoracic Cobb angle, Thoracolumbar Cobb angle and Lumbar Cobb angle are the dependent variables. Rib-index (thoracic deformity) is the independent variable. The only linear association was the one between Thoracic Cobb Angle and rib-index in the age group of 14-18 years. (*Predicted Thoracic Cobb Angle = -6.357 + 7.974 × (Rib-Index)*.

Therefore the age is a very important factor and has a definite effect, since it influences the correlation between the surface and the spinal deformity. In younger children this correlation is very weak, while it is stronger in older children. This important finding of the existence of remarkable rib cage deformity without simultaneous spinal deformity in younger school screening referrals is a fact that requires further research. A longitudinal study ought to be conducted to discriminate the percentage of children that will in time develop scoliosis and the possible responsible factors. As a result of the effect of growth on the correlation between the thoracic surface deformity and the spinal deformity, the predictive value of the existing formulas which calculate the Cobb angle from surface measurements is poor. Therefore the recommendation is to take into consideration the effect of growth when developing such predictive models, otherwise they can be inaccurate [12] (Fig. 2).

The angle measured by a scoliometer does not correspond to the Cobb angle measured on a radiograph [13]. Furthermore the Cobb angle alone cannot explain the whole of the surface deformity [14]. As a consequence, not all patients with radiographic scoliosis have rotation of the trunk, and not all patients with trunk rotation have radiographic scoliosis [15]. Goldberg [70] and Kotwicki [69] agree that "surface parameters corresponding with radiological ones are neither possible nor expedient as both methods focus on different aspects of the deformity. The 3D presentation accompanied by numerical data that is produced in surface topography offers a more complete perspective of the deformity of the back surface and enables a more thorough analysis of the patient's deformity pattern".

### The Cobb angle

The degree of curvature in the coronal plane is radiographically measured according to the method of Cobb [16]. The Cobb angle, which is considered the golden standard, is the angle between lines drawn along the upper end plate of the most tilted vertebrae above the curve's apex and the lower end plate of the most tilted vertebrae below the apex. While Cobb angle is the accepted standard for measuring scoliosis on radiographs [17,18], it has some important limitations [10,18]:

▪ The Cobb angle describes only one plane of the 3D deformity.

▪ The Cobb angle is not linearly proportional to the severity of scoliosis in a linear fashion (ie, a curve with a Cobb angle of 40° is more than twice as severe as a curve with a Cobb angle of 20°).

▪ Cobb angle measurement has a reported intra-observer variability of 2.8°-4.9° and an inter-observer variability of 6.3°-7.2° [19,20] when traditional techniques are used. Recent advances in measurements on digitally acquired radiographs provide far more accurate results, with a reported intra-observer and inter-observer variability of 1.3° [21].

Back surface mapping for scoliosis screening has been used for many years as a valid alternative to either use of x-rays or scoliometer measurements. From the beginning it became clear that "Because surgeons are so familiar with Cobb angle measurements on radiograph, the introduction of new surface shape measures whose meaning may not be readily apparent to clinicians has been difficult" [22]. This explains the effort over the years to relate surface shape parameters with Cobb angle [eg. [23-25,71]].

However, over the years it became apparent that the Cobb angle measures only one aspect of the 3D deformity and that the correlation between the Cobb angle and the surface parameters is negligible [26,6,27]. However, it is noted that the more severe the Cobb angle the more the surface deformity is pronounced.

Lately, many researchers are seriously questioning the effectiveness of such efforts and strong statements, like this appear "Searching for relationship between radiological Cobb angle and surface parameters with making presumption that the higher correlation with Cobb angle, the better the surface technique may be one of the reasons that introduced the surface topography in a blind alley. In fact, Cobb angle is nothing more than a shadow of two limit vertebrae. It is not clear what would be the rationale to expect that so constructed angle should highly correlate with any of the surface describing parameters." [28]. And as Kotwicki states it "When debating on the role of the surface topography in the evaluation of the body morphology in children with idiopathic scoliosis, one should begin with rejecting the dogma of the radiological Cobb angle, as the only gold standard for scoliosis evaluation. " [77].

### Optical techniques

Optical systems have been developed as non-invasive imaging techniques. Examples of such systems are the Moiré-fringe mapping [29], the structured light techniques like the Integrated Shape Imaging System (ISIS) [30-34], or the Quantec system [35,14,37] or the Ortelius [18] scanners, and devices that scan 360° torso profiles [38-41], ultrasound systems [42], 3D body scanners (eg. Inspeck, Cyberware, TC2, Minolta Vivid, Vitus 3D, etc) [2], the Formetric video-raster-stereography system http://www.diers.de[72-76] and last but not least stereo-photogrammetric systems [43-47].

Regarding moiré topography, since Takasaki [29] first introduced it, many other researchers [48-52] have effectively used this technique. Regarding Moiré the following conclusions are useful to our discussion [50]:

▪ There is no correlation between Moiré asymmetry and the Cobb angle

▪ The risk of obtaining false negatives is low (ie. high sensitivity)

▪ The risk of obtaining false positives is high (ie. low specificity)

### Metrics in scoliosis evaluation

In a vast related medical literature, one can find quite a few scoliosis evaluation indices, which are based on back surface data and are generally measured along the three planes (coronal, transverse and sagittal). However, there exist no coherent presentation of the underlying metrics, the involved anatomic surface landmarks and the definition of the planes and the related body axes they refer to.

Generally speaking, the scoliosis parameters which have been used up to now belong to one of the following groups: (a) the first group includes indices which are specific to the measurement technique. These indices depend on the measurement technique, which means that cannot be measured and by other means. Such examples are eg. the angles q1 and q2 in QSIS which are angles formed by the tangents to the corresponding fringes in the Moiré system. Obviously, theses cannot me measured with other means than moiré. (b) The second group are indices independent of the measuring technique. This makes them more useful, since they can be used to evaluate scoliosis given that the back surface topography is known in 3D, regardless of the measuring techniques used. Such example is eg. the Angle of Trunk Rotation index, which can be evaluated by scoliometer measurement, by moiré techniques or by any other 3D surface measurement.

After many years of research and discussion, in 2009 the International Society on Scoliosis Orthopaedic and Rehabilitation Treatment (SOSORT) reached to important agreements among their members as published in the 6^th ^SOSORT consensus paper [28]. Although the agreements/conclusions concern a number of issues related to scoliosis (see Table 1), for the economy of this paper only the issues related to back surface measurements are highlighted next:

**Table 1:** Scoliosis surface parameters after 6^th ^SOSORT consensus paper

No.	Conclusion	Item	Agreement
**1**	Position/view of the patient for surface topography measurement [table eighteen]	Position: standing upright	100%
		
		View: Back	100%

**2**	Anatomic surface landmarks to be taken into consideration systematically [table nineteen]	Spinous processes	100%
		
		Posterior iliac spines	100%
		
		Shoulders	100%
		
		Scapulae	88.9%

**3**	Surface parameters recommended for systematic use [table twenty]		

3.1	Body axis definition	Analogous to radiological VCSL	100%

3.2	Frontal plane analysis	Curve angle	75%
		
		Shoulders	66.7%
		
		Scapulae	66.7%

3.3	Sagittal plane analysis	Relation of C7 to S1	100%
		
		Cervical lordosis	100%
		
		Thoracic kyphosis	100%
		
		Lumbar lordosis	100%

3.4	Transverse plane analysis	Trunk rotation main curve	100%
		
		Trunk rotation Compensatory curves	100%

3.5	Pelvis	PSIS height	100%

**4**	Further Conclusions	Scoliometer ATR measure for transverse plane deformity	95%
		
		Cobb angle measurement as radiological parameter	100%

## The reference planes

**Figure 3:**
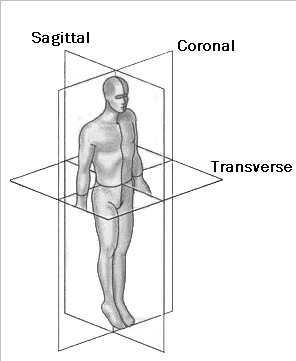
**The three reference planes used for scoliosis parameters**.

There is a wide agreement and usual practice over the years to use the three mutually perpendicular planes (Coronal, Sagittal and Transverse) as reference to scoliosis parameters (Fig. 3). There is no reference in the literature of any other reference frame in use.

## Anatomic surface landmarks

In order for any measurement taken at different times to be mutually comparable, either the involved metrics should be coordinate-free or they should refer to the same coordinate system.

The first case is rather rare and refers to metrics like areas, volumes, etc. The second case is the usual case and mainly refers to coordinates, angles, distances and the like. In this latter case there is a need to establish a coordinate system, which is stable between the screening sessions.

**Figure 4:**
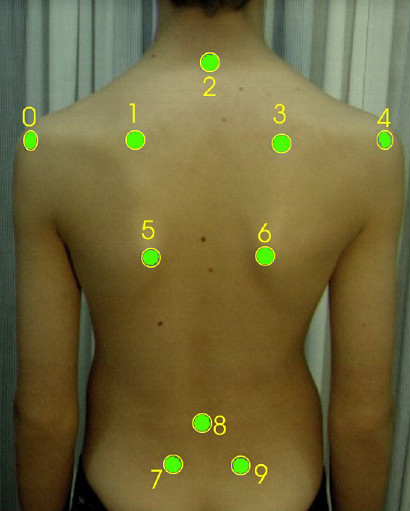
**Anatomic landmarks used for back surface measurements as suggested by SOSORT**. 2: Spinous process of C7, 8: Spinous process of L4, 0, 4: Acromial Angle of shoulders, 1, 3: Superior Angle of Scapulae, 5, 6: Inferior Angle of Scapulae, 7, 9: PSIS-Posterior Superior Iliac Spine.

Any attempt to establish such a constant system through points on the background creates major technical problems and is cumbersome in use. The only vital solution is to use a "body specific" coordinate system, in which case stable anatomical landmarks are necessary. The SOSORT consensus shows 10 such points (see SOSORT conclusion No. 2), which are depicted in Fig. 4.

**Figure 5:**
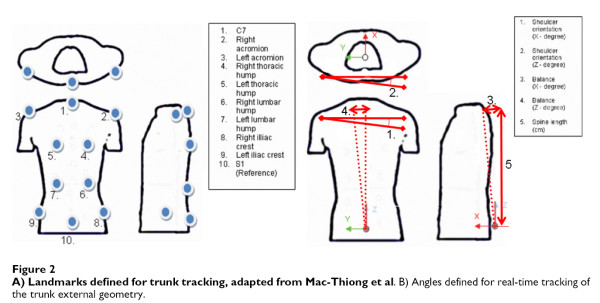
**Anatomic landmarks used in **[54].

**Figure 6:**
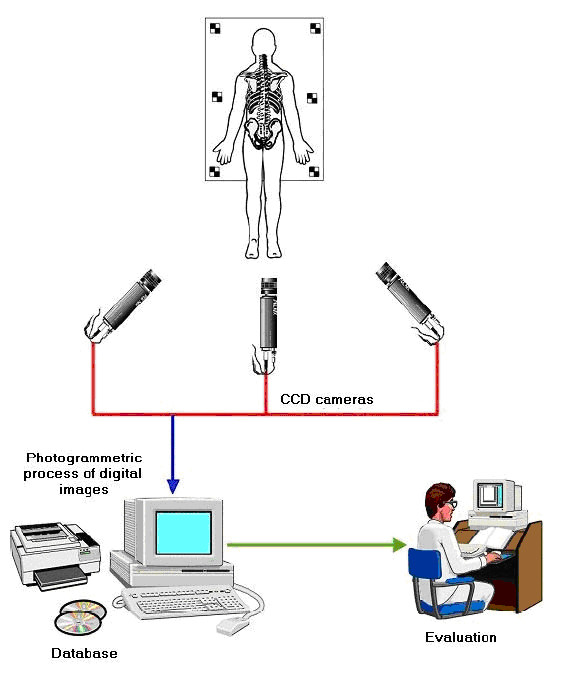
**Reference frame landmarks used in **[47].

The same anatomical landmarks have been used by many researchers, as for example [53,54,47] (Fig. 5, 6). The Integrated Shape Imaging System (ISIS) [30] uses also the C7/T1, the PSIS (Posterior Superior Iliac Spines) points and their point the sacrum, and a sufficient number of spinous processes.

**Figure 7:**
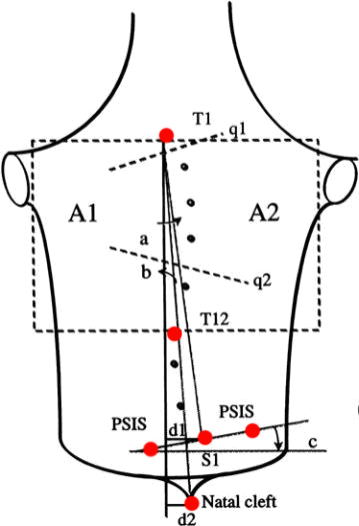
**Anatomic landmarks used in QSIS (after **[55]**)**. T1: Spinous process of T1, T12: Spinous process of T12, S1: Spinous process of S1m, NC: Natal Cleft, PSIS 1,2: PSIS-Posterior Superior Iliac Spine. Note: q1 and q2 angles can only be measured with the moiré fringes.

Similar, but not exactly the same, landmarks have been used by other systems, eg. in QSIS (Fig. 7). The Quantec Spinal Image System (QSIS), is based on raster stereography [33,55]. QSIS uses color markers of a diameter of 6.0 mm, which are attached to each spinous process from T1 to L5, including the two PSIS. The multiple fringes are projected onto the surface of the back above the natal cleft. A total of 12 metrics are produced from the 3D surface data.

**Figure 8:**
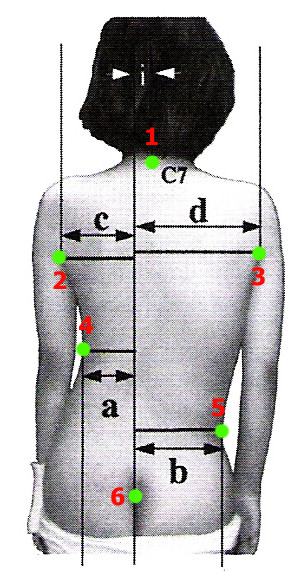
**Anatomic landmarks used in POTSI index (after **[56]**)**. 1: Spinous process of C7, 2: Left Axilla fold, 3: Right Axilla fold, 4: Most intended point of the Left Trunk, 5: Most intended point of the Right Trunk, 6: Natal Cleft (NC). Note: All horizontal distances are measured from the Vertical line passing through the NC point.

In contrast, another popular index, the POTSI index [56] (Posterior Trunk Symmetry Index) (Fig. 8) is using the axilla folds and the most intended waist points as landmarks.

**Figure 9:**
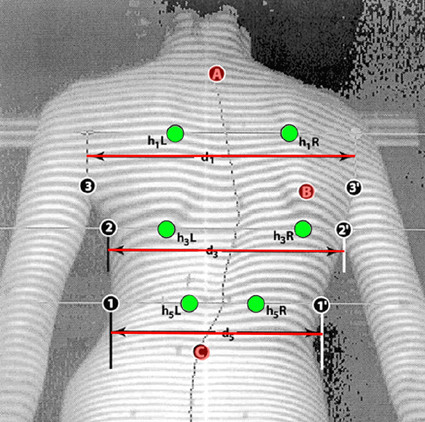
**Anatomic landmarks used in SHS index (after **[56,57,68]**)**. A: Spinous process of C7, B: the max. prominence of the angle of the scapula, C: the lowest indentation of the lumbar lordosis, h_1_L - h_1_R = Height difference of Left and Right low and high (anterior and posterior) point at Thoracic level, h_3_L - h_3_R = Height difference of Left and Right low and high point at Thoraco-Lumbar level, h_5_L - h_5_R = Height difference of Left and Right low and high point at Lumbar level.

**Figure 10:**
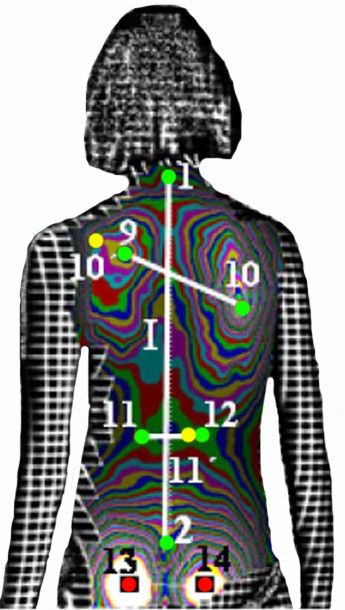
**Anatomic landmarks used in DAPI index (after **[59]**)**. 1: Spinous process of C7, 2: top of the intergluteal furrow, 9: most prominent point of the left scapula, 10: most prominent point of the right scapula, 11: least prominent point of the waist line, left, 12: least prominent point of the waist line, right, 10': symmetric point of 10 on line 10-9, 11': symmetric point of 11 on line 11-12. Note: Points 13 and 14 (most prominent point of the left and right gluteus) are used to correct, if necessary, the incorrect placement of the patient. Points 13 and 14 must have equal prominence if the patient is positioned correctly.

In Fig. 9 and 10 the landmarks used by the SHS [56-58,68] (Suzuki Hump Sum) and the DAPI indices [59] (Deformation of the Axial Plane Index) are given.

## Body coordinate system

**Figure 11:**
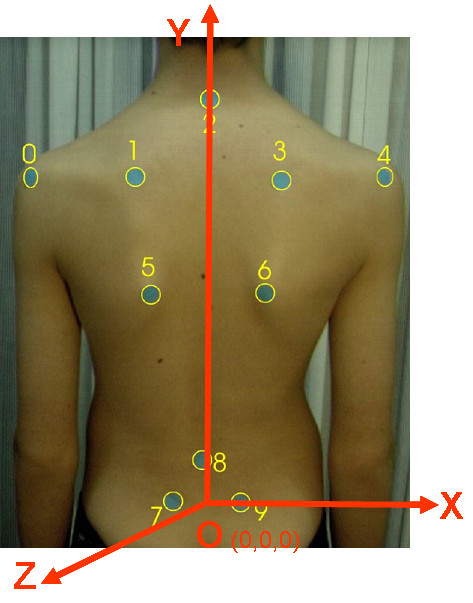
**The usually adopted coordinate system**. O:The origin of the Body Coordinate System. It is defined as the midpoint of the line 7-9, Y axis: (VCSL-Vertical Central Sacral Line) Vertical line passing through O, X axis: Horizontal line passing through O, Z axis: Forms with X a horizontal plane (see also SOSORT Conclusion 3.1).

**Figure 12:**
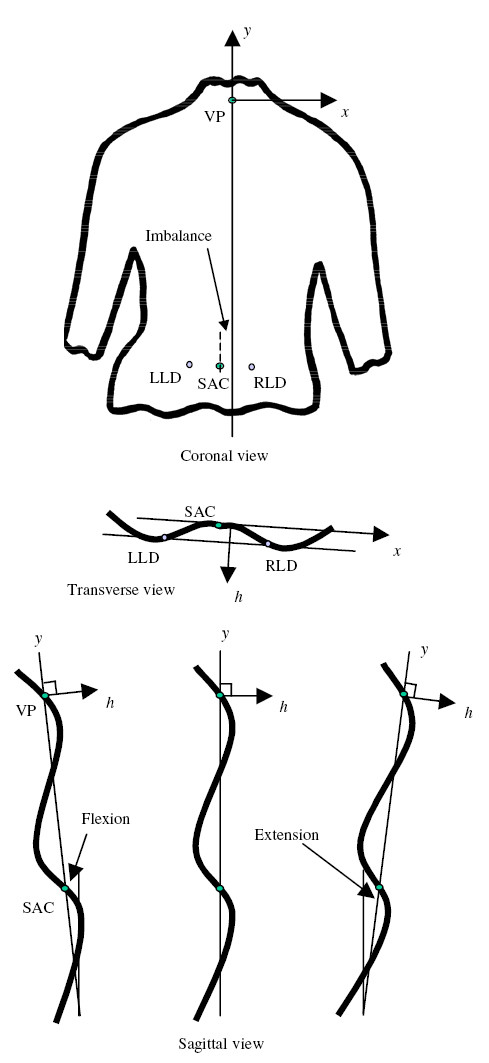
**The used coordinate system in **[34].

**Figure 13:**
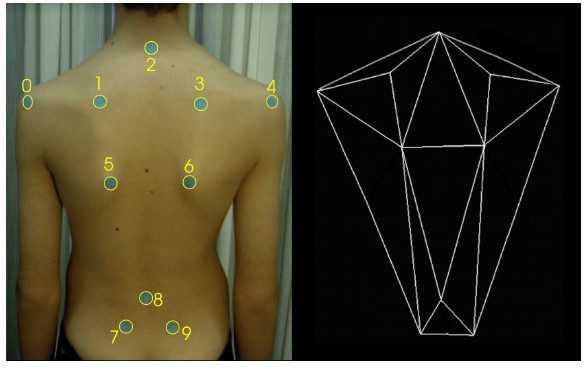
**The used coordinate system in **[53].

The coordinate system usually adopted is shown in Figure 11. Many researchers [53,34,54,47] (Fig. 12, 13) prefer such a body system simply because it can be easily established, since it is based on sound body landmarks, which are easily traceable and marked by the physician.

The VCSL (Vertical Central Sacral Line) line is also used in QSIS system, in POTSI and DAPI index definition, etc., while the Z axis definition is compatible to that used in SHS and DAPI (see section 6).

## Scoliosis deformity indices: A literature survey

One can find quite a number of scoliotic indices in the literature. Here, for methodological reasons, we are going to present them grouped by the plane they refer to. The reason for such a presentation is twofold: first, to present them in a logical way according to the type of deformity they are able to measure; and secondly to lead the discussion to the degree of correlation existing among them.

### Deformity indices measured on the Coronal plane

**Figure 14:**
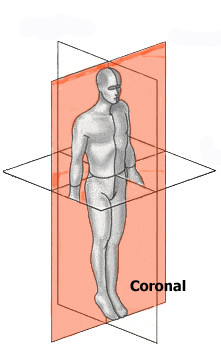
**Major deformity indices measured on the Coronal plane**. 1. Cobb angle, 2. Spinous process line [60], 3. Nault indices [3], 4. Shoulder orientation Balance [54], 5. ISIS2 Lateral index [34], 6. POTSI Index [56,61], 7. QSIS indices in the Coronal plane [55], 8. ASY1, Z1, Z2 [47], 9. Body curve, Head rib pelvis, Head pelvis, Shoulder level, Scapula rotation WRVAS qualitative [23], 10. TRACE qualitative Indices [27]. (see also SOSORT Conclusion 3.2, 3.5, 4).

**Figure 15:**
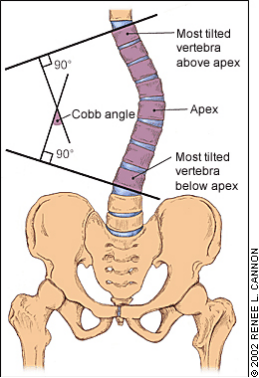
**The Cobb angle**.

Coronal plane is the major plane for measuring back deformity (Fig. 14), since it is related to Cobb angle (Fig. 15) definition. Since Cobb angle can be obtained only with x-ray measurements, back surface indices were invented to simulate the Cobb angle.

The spinous process line of Jaremko [60] and the similar but qualitative indices used in WRVAS (Walter-Reed Visual Assessment Scale) [27,24] belong to this logic line. Similar to them are also the ASY1 index of [47] as well as the Integrated Shape Imaging System (ISIS2) LA (Lateral Asymmetry) index [34]. In the latter, a 5^th ^order polynomial is fitted through the spinous process line (as depicted by 19 transversal sections).

**Figure 16:**
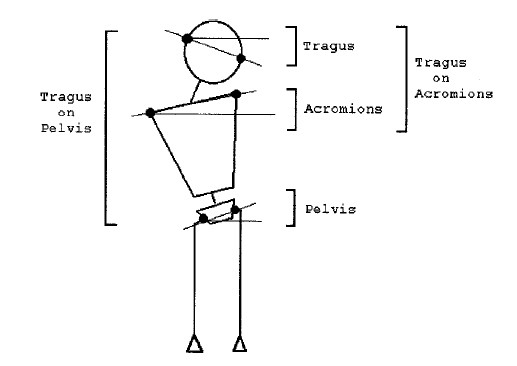
**Deformity indices measured on the Coronal plane by Nault et al (after **[3]**)**.

**Figure 17:**
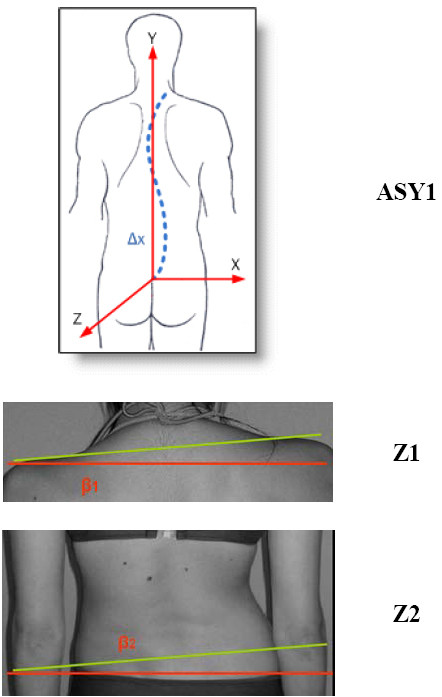
**Deformity indices measured on the Coronal plane by Patias et al (after **[47]**)**.

On the other hand, the indices suggested by Nault et al. [3] (Fig. 16), [54] and [47] (Fig. 17) use the landmarks of shoulders and scapula to measure the body balance, following thus the SOSORT consensus conclusions.

**Figure 18:**
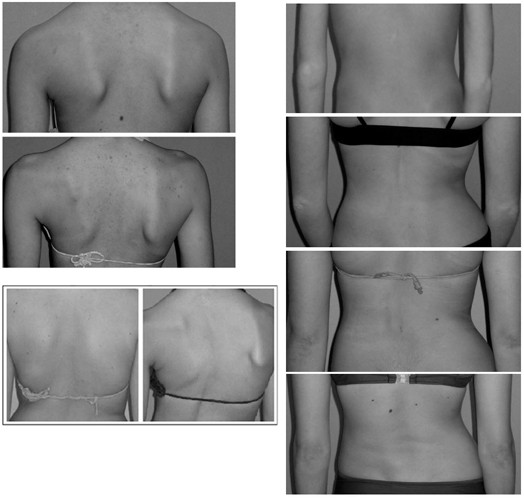
**TRACE Indices after **[27].

Asymmetries in shoulders, scapulae, waist and hemi-thorax have been used also in the TRunk Aesthetic Clinical Evaluation (TRACE) tool [27] (Fig. 18), which is also qualitative.

**Figure 19:**
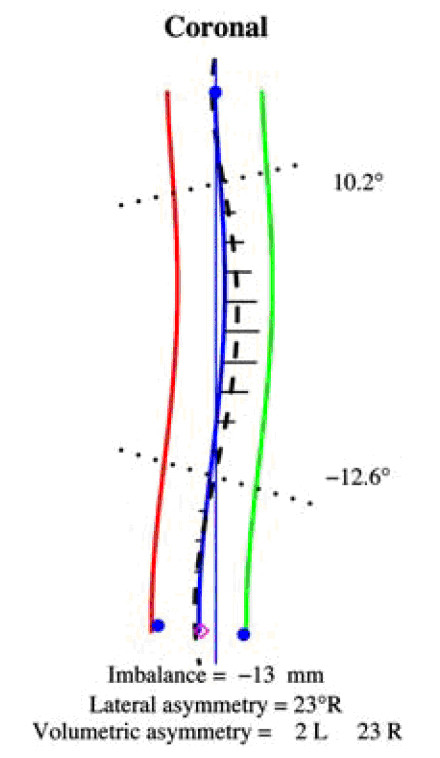
**ISIS LA index after **[34].

**Figure 20:**
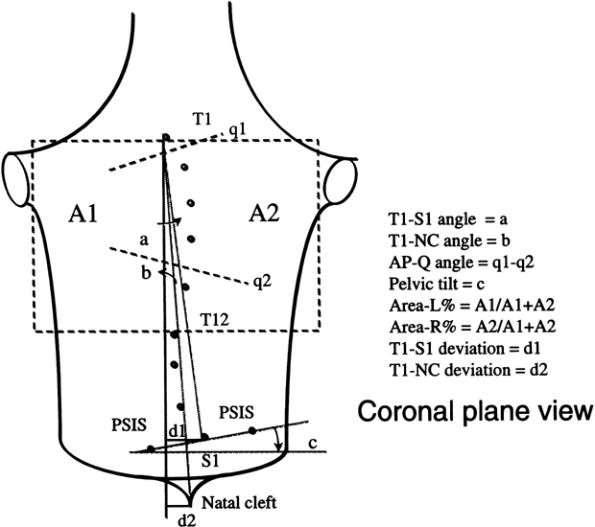
**QSIS indices in the Coronal plane after **[55]. a: angle between the vertical and the line T1-S1, b: angle between the vertical and the line T1-natal cleft, c: angle between the horizontal and the PSIS line, d1: horizontal distance of S1 from the vertical, d2: horizontal distance of the natal cleft from the vertical, q1: Max. tilt line. q1 is plotted as tangent to homologous moiré fringes, q2: Pelvic tilt angle. Plotted in a similar to q1 manner, AP-Q: q1 - q2, Area-L or R %: Area percentage of left or right: each lateral back area divided by the total back area as defined from T1 to T12.

The ISIS system uses the Imbalance, Lateral Asymmetry and volumetric asymmetry Indices (Fig. 19) as indices in the Coronal plane, while the QSIS system uses a series of angles and distances (Fig. 20) for the same reason.

**Figure 21:**
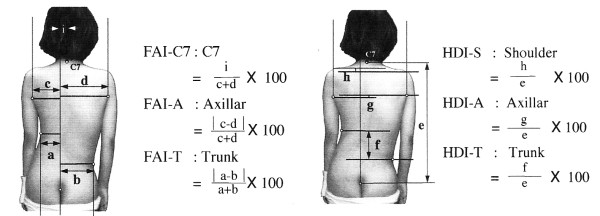
**POsterior Trunk Symmetry Index (POTSI) after **[56,61]. The POsterior Trunk Symmetry Index (POTSI) is computed as a sum of the 6 indices: POTSI = (FAI-C7 + FAI-A + FAI-T) + (HDI-S + HDI-A + HDI-T).

POTSI (Fig. 21) is another popular composite index of the coronal plane. It actually consists of 6 sub-indices, three of which measure the asymmetry along the X axis and the other three along the Y axis.

### Deformity indices measured on the Transverse plane

**Figure 22:**
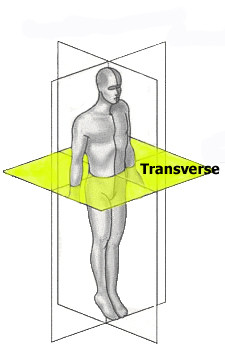
**Major deformity indices measured on the Transverse plane**. 1. Angle of Trunk Rotation (ATR, or ATI - Angle of Trunk Inclination) [62], 2. CTAS [63,64], 3. Torso centroid line, Principal axis orientation, Back surface rotation, Envelope indices, Half-area indices, Quarter-area indices [60], 4. Rib prominence, Flank prominence [27], 5. ISIS2 TA (Transverse Asymmetry index), VA (Volume Asymmetry index) and HS (Hump Severity index) [30,33,34], 6. Suzuki Hump Sum (SHS) [68], 7. Deformity in the Axial Plane Index (DAPI) [59], 8. QSIS indices in the Transverse plane [55], 9. Y1, ASY2, ASY3 [47] (see also SOSORT Conclusion 3.4 and 4).

**Figure 23:**
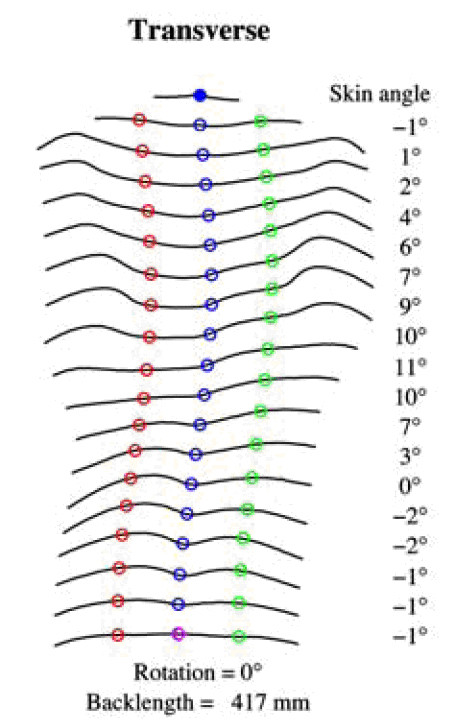
**ISIS TA, VA, HS after **[34,33].

Transverse plane is the second major plane for measuring back deformity (Fig 22), since it is related to Adams test. The major measurement refers to scoliometer and the major index used with reference to this plane is the "Angle of Trunk Rotation" (ATR, or ATI - Angle of Trunk Inclination) [62]. Very similar to ATR is the ISIS2 Transverse index [34] (Fig. 23), where the shape of the transversal section is computed for 19 equally spaced sections.

**Figure 24:**
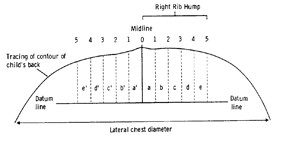
**CTAS index after **[63]. *Crude Trunk Asymmetry Score (CTAS)*: CTAS = (a-a')+(b-b')+(c-c')+(d+d')+(e-e').

**Figure 25:**
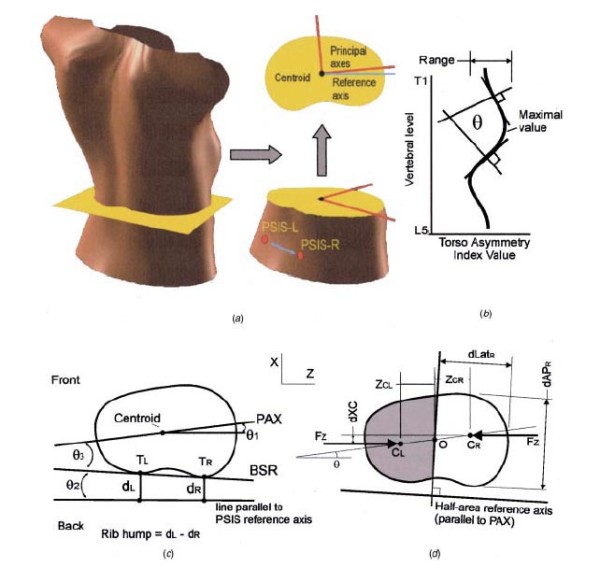
**Transversal Indices after **[60].

**Figure 26:**
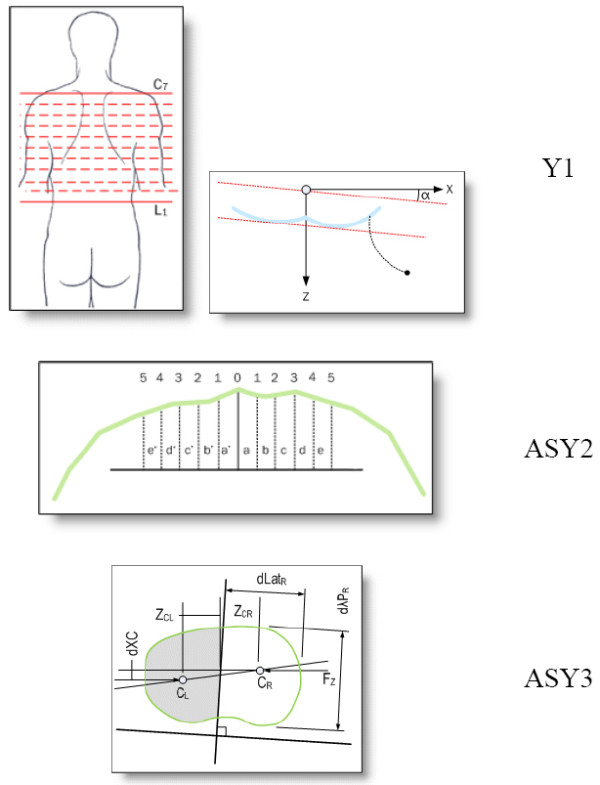
**Transversal Indices after **[47].

**Figure 27:**
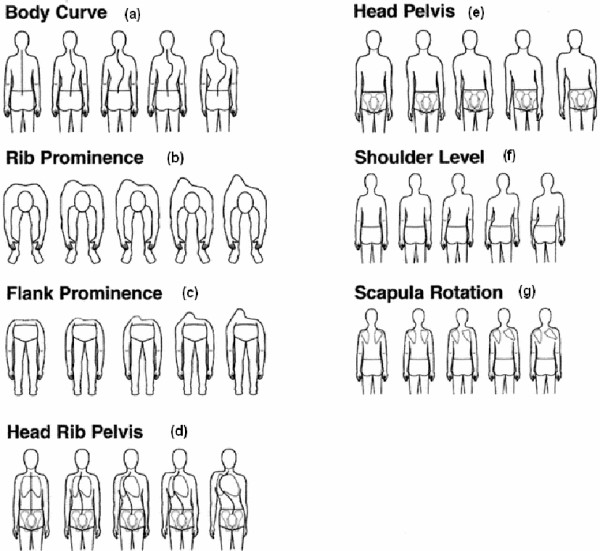
**Transversal Indices after **[27].

**Figure 28:**
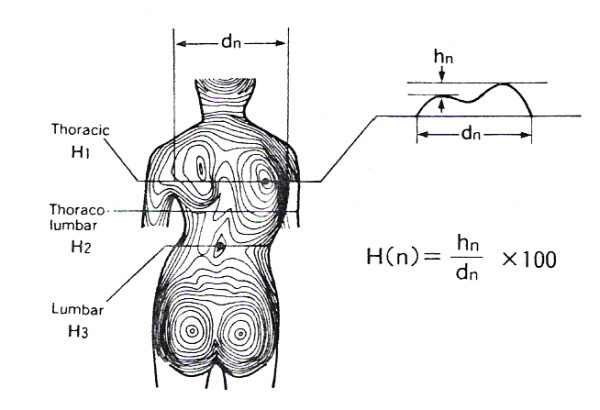
**Suzuki Hump Sum (SHS) after **[68]. SHS = HIX1 + HIX3 + HIX5.

**Figure 29:**
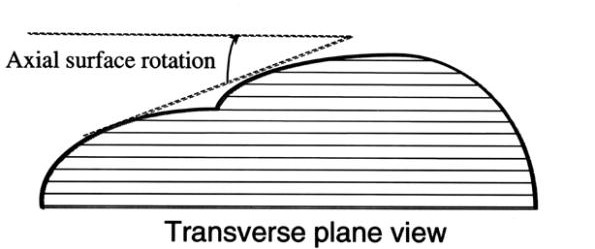
**QSIS indices in the Transverse plane after **[55].

**Figure 30:**
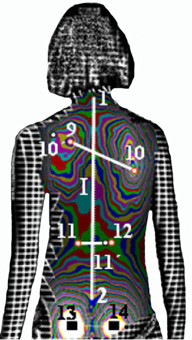
**DAPI index after **[59].

Besides ATR, the Crude Trunk Asymmetry Index (CTAS) index [63,64] (Fig. 24) is emulating the "formulator body contour tracer" measurements, while other indices, like those suggested by Jaremko [60] (Fig. 25) and Patias [47] (Fig. 26) are duplicates of the above in the general sense. The Sanders suggestions [27,24] (Fig. 27) are related to the WRVAS test which is only of qualitative nature. A popular transversal index is the SHS (Fig. 28) index, which measures the hump height difference at three sections and adds up the relative sub-indices. Kotwicki [69] raises concerns on whether SHS measurements at three levels only are adequate and he suggests an improvement to SHS, namely the SoR. SoR (Sum of Rotation) index adds up measurements at 17 vertebra (12 thoracic and 5 lumbar). The QSIS axial surface rotation (Fig. 29) is simply the ATR measured by the scoliometer, while the DAPI index (Fig. 30) measures the minima and maxima height differences of the trunk points.

### Deformity indices measured on the Sagittal plane

**Figure 31:**
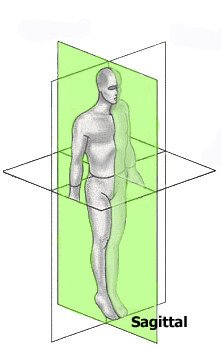
**Major deformity indices measured on the Saggital plane**. 1. Relation of C7 to S1, 2. Cervical Lordosis, 3. Thoracic Kyphosis (TK), Lumbar Lordosis (LL) [Fleche method, [65], 4. ISIS2 Saggital index [34], 5. Nault indices [3], 6. QSIS indices in the Sagittal plane [55], 7. Kyphosis and Lordosis indices [66] (see also SOSORT Conclusion 3.3).

**Figure 32:**
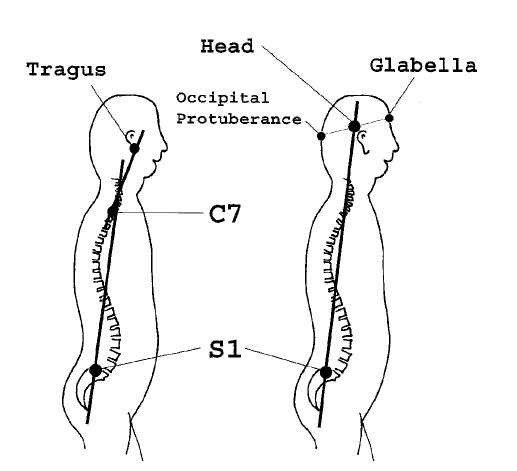
**Sagittal Indices after **[3].

**Figure 33:**
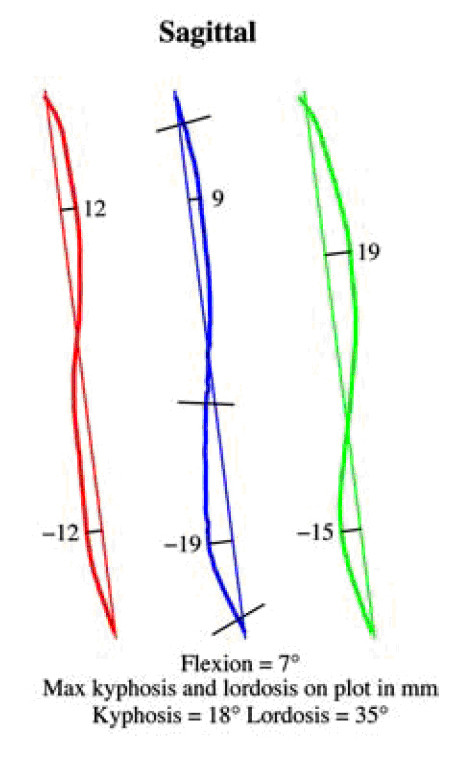
**ISIS2 indices after **[34].

**Figure 34:**
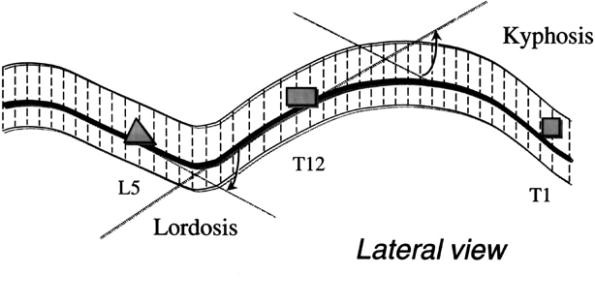
**QSIS indices in the Sagittal plane after **[55].

**Figure 35:**
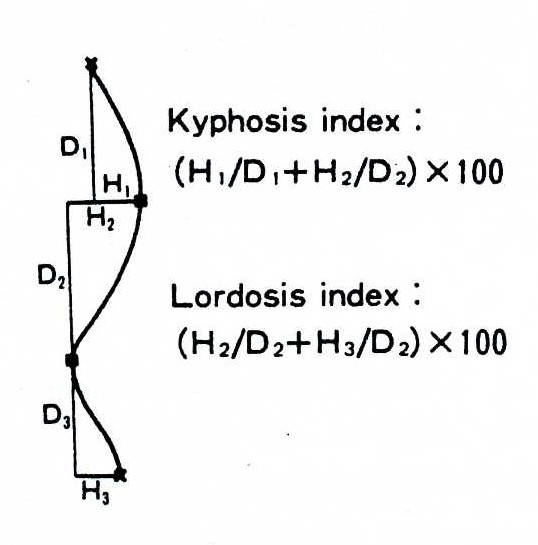
**Kyphosis and Lordosis indices after **[66].

**Figure 36:**
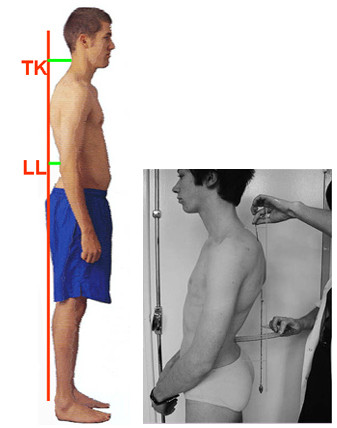
**Thoracic Kyphosis (TK), Lumbar Lordosis (LL), Fleche method **[65].

Sagittal plane is the least used plane for referring back deformity (Fig. 31). Actually there are very few indices in the literature, which are computed in this plane. Mainly the Nault [3] (Fig. 32), the ISIS2 indices [34] (Fig. 33), the QSIS indices (Fig. 34) and the Sinoto indices (Fig. 35) are referring to the location and the magnitude of the maximum Kyphosis and Lordosis [66]. To these indices also there is a consensus by SOSORT. Additionally, measuring techniques for kyphotic deformities are defined also by the Fleche-method [65] (Fig. 36).

## Discussion and Conclusions

Understanding scoliosis or other trunk deformity is a complex issue since it evolves in three dimensional space. Many technologies have been developed and used over the years and each technology offers new approaches in understanding and describing scoliosis through different sets of indices. Out of this massive data the scientific society has to choose measures and define methodologies in order to optimally diagnose, quantify, document and assess the progression of scoliosis for both clinical treatment and cosmetic improvement.

After all these years of research it is apparent that for trunk deformity description a single value index is not adequate. Unfortunately, currently, a general consensus on a set of indices does not exist, and this makes this review useful. Our effort is a clear presentation of the proposed indices over the years, in a way that productive conclusions can be reached:

1. It is clear now that surface metrics have very little correlation to Cobb angle measurements (eg. [56] regarding POTSI index). In addition, it has also been reported that patients with double curves have significantly less trunk deformity in both the transverse and coronal plane than patients with thoracic and thoraco-lumbar curves of similar Cobb size [57].

2. It should also be clear that indices measured on different planes do not correlate to each other. Examples are Cobb angle vs. Scoliometer angle, Cobb vs. Rib and Flank prominence, etc.

3. Different indices exhibit quite diverging characteristics in terms of observer-induced errors, accuracy, sensitivity and specificity. Although a complete comparison can not be found in the literature, tabularizing the results and conclusions given by different researchers [56,58,59], we give below (Table 2) the specifics for different popular indices.

**Table 2:** Characteristics (observer-induced errors, accuracy, sensitivity and specificity) of different popular indices

	Intra-observer error	Inter-observer error	Threshold for scoliosis cases	**Threshold for change (as suggested by Asher **[58]**)**	Sensitivity	Specificity
**Cobb**	4°	7°		± 5°	High	Low

**POTSI**	5.5	6.4	28.1	± 8	Low	High

**SHS**	1.2	1.9	9.0	± 3.5		

**DAPI**					High	Low

**Moiré**					High	Low

**Adam**			0°		High	Low

It is clear that complicated positioning of the patient and ambiguous anatomical landmarks are the major error sources, which cause observer variations. For instance, moiré techniques generally suffer from errors due to malpositions of the patient and generally require strict and cumbersome protocols for positioning the patient. "A major drawback of moiré topography is that while the shape information is displayed, it is not in a form which can be unambiguously interpreted" [30]. POTSI index is reported [59] to introduce errors due to the difficulty in situating the points involved for calculating the index, as some of them are located in the shaded areas, while they are not anatomical points easily and uniquely identifiable. "The ISIS system lacked accuracy mainly because of the difficulty of distinguishing adequate landmarks due to shadowing effect" [67].

Therefore, based on the experience gained from this extended literature review, we think it is useful to lay down the principles that should be followed when an index is proposed.

### Principles for optimally designed scoliosis Indices

1. ***Indices should be measured with the maximum achievable accuracy and in a direct manner***. For instance, Coordinates and Angles are direct measurements whereas areas, volumes etc. are indirectly calculated from other direct measurements. Therefore indices based on direct measurements are more accurate and should be preferable.

2. ***Indices should be independent from the method of measuring the back surface deformities***. If this is not the case then indices can not be of universal use, and will also highly depend on the current technology.

3. ***Indices should be based on robust procedures and automatic measurements and should be evaluated by automatic processing techniques***, eliminating as far as possible the human intervention. The reported levels of inter-/intra-observer variability and accuracy of the indices used so far reveals this problem. Only with automation the observer variability, the human induced errors, objectivity, and required experience will be eliminated.

4. ***Indices should be based on automatically detectable and uniquely identifiable anatomical landmarks***. This is closely connected to point No. 4 above. Both the landmarks used and the measured points on the back surface should be unambiguously positioned, properly signalized and automatically detected and measured on the image.

5. ***Indices should require simple measuring protocols***. Complicated or demanding protocols are sources of errors. This includes also (and especially) patient position and orientation relative to the sensor, lighting conditions, etc. Indices should be independent from and robust with respect to these parameters as much as possible.

6. ***Indices should be normalized in order to be comparable among patients***. This means that the indices should not depend on the trunk size, on the width of the waist or the length of the arms. In this respect, indices should be unitless, percentages etc.

7. ***Indices should provide a stable datum for progress monitoring over time***. This means that indices should either be coordinate-system-free of refer to a coordinate system which is stable over time.

8. ***Indices should be able to distinguish between different types of surface deformities***, ie. Coronal/Transverse/Sagittal, Left/Right semi-trunk, Thoracic/Thoraco-Lumbar/Lumbar, Single/Double curves.

9. ***Indices should provide a clear and safe difference in magnitude between normality and pathology***, so that pathology can be safely distinguished and diagnosed. This actually means increased sensitivity and specificity. It also means that the indices should have small typical error relative to the smallest change (progression) we would like to detect.

## Competing interests

The authors declare that they have no competing interests.

## Authors' contributions

All authors have contributed in literature search and review. PP and TBG drafted the text.

All authors read and approved the final manuscript.
